# SoNAC72-SoMYB44/SobHLH130 module contributes to flower color fading via regulating anthocyanin biosynthesis by directly binding to the *SoUFGT1* promoter in lilac (*Syringa oblata*)

**DOI:** 10.1093/hr/uhae326

**Published:** 2024-11-21

**Authors:** Jinxuan Wang, Xin Wang, Bo Ma, Pingsheng Leng, Jing Wu, Zenghui Hu

**Affiliations:** Engineering Research Center for Ancient Tree Health and Ancient Tree Culture of National Forestry and Grassland Administration, College of Landscape Architecture, Beijing University of Agriculture, 7# Beinong Road, Beijing 102206, China; Engineering Research Center for Ancient Tree Health and Ancient Tree Culture of National Forestry and Grassland Administration, College of Landscape Architecture, Beijing University of Agriculture, 7# Beinong Road, Beijing 102206, China; Engineering Research Center for Ancient Tree Health and Ancient Tree Culture of National Forestry and Grassland Administration, College of Landscape Architecture, Beijing University of Agriculture, 7# Beinong Road, Beijing 102206, China; Engineering Research Center for Ancient Tree Health and Ancient Tree Culture of National Forestry and Grassland Administration, College of Landscape Architecture, Beijing University of Agriculture, 7# Beinong Road, Beijing 102206, China; Beijing Laboratory for Urban and Rural Ecological Environment, Beijing University of Agriculture, 7# Beinong Road, Beijing 102206, China; Engineering Research Center for Ancient Tree Health and Ancient Tree Culture of National Forestry and Grassland Administration, College of Landscape Architecture, Beijing University of Agriculture, 7# Beinong Road, Beijing 102206, China; Beijing Engineering Research Center of Rural Landscape Planning and Design, Beijing University of Agriculture, 7# Beinong Road, Beijing 102206, China; Engineering Research Center for Ancient Tree Health and Ancient Tree Culture of National Forestry and Grassland Administration, College of Landscape Architecture, Beijing University of Agriculture, 7# Beinong Road, Beijing 102206, China; Beijing Engineering Research Center of Rural Landscape Planning and Design, Beijing University of Agriculture, 7# Beinong Road, Beijing 102206, China

## Abstract

The fading of flower color is caused by changes in anthocyanin content during flower development in many plants, including lilac (*Syringa oblata*). However, the molecular regulatory mechanism of this phenomenon is still poorly understood. UDP-glucose: flavonoid 3-*O*-glucosyltransferase (UFGT) has a pivotal role in the formation of stable anthocyanins. Here, *SoUFGT1* and three transcription factors, SoMYB44, SobHLH130, and SoNAC72, were identified and verified to participate in anthocyanin production in lilac. Overexpressing *SoMYB44* promoted *SoUFGT1* expression in lilac petals. The yeast one-hybrid (Y1H) and dual-luciferase (Dual-LUC) assays demonstrated that SoMYB44 activated *SoUFGT1*, thereby bolstering anthocyanin accumulation. The overexpression and silencing of *SoNAC72* in petals revealed that it facilitated anthocyanin accumulation. The Y1H and Dual-LUC assays verified that SoNAC72 was capable of directly binding to the *SoMYB44* promoter to activate the latter's expression. In addition, SobHLH130 was also displayed to mediate anthocyanin accumulation in petals. By using yeast two-hybrid (Y2H) and bimolecular fluorescence complementation (BiFC) assays, the interaction between SoMYB44 and SobHLH130 was confirmed. These results corroborated that SoNAC72 regulates *SoMYB44* expression, and SoMYB44 interacts with SobHLH130 to trigger *SoUFGT1* expression in lilac, which then contributes to their anthocyanin accumulation. In sum, along with lilac flower development, the lower expression of *SoNAC72* and *SobHLH130* reduces *SoMYB44* transcripts and depresses transcriptional regulation of *SoUFGT1*, thus diminishing anthocyanin biosynthesis, leading to the fading of petal color. These study's findings provide valuable new insight for understanding the formation and regulatory mechanisms of flower color in lilac.

## Introduction

Flower color confers horticultural plants' outstanding ornamental value and has major ecological functions, such as attracting pollinators, in addition to being crucial for the breeding of new cultivar [1]. Moreover, flower color is known to change regularly during development. The color of some flowers changes from light to dark during blooming, such as *Pleroma raddianum* [2] and *Combretum indicum* [3]. In contrast, many flowers are darker in color during the early development periods and then fade later on [4, 5]. However, the regulatory mechanisms involved are poorly known, which has hindered an understanding of the ecological functions and molecular breeding of flower color.

Lilac (*Syringa oblata*) is a representative plant of the Oleaceae family that is native to China, and it is broadly distributed in different temperature zones [6]. As a traditional and popular ornamental woody plant with strong adaptability during early spring, lilac has been planted for thousands of years in China [7]. Its purple flowers rich in anthocyanins are an important ornamental feature, and this purple color fades from the bud stage through the full-opening stage during the flower development process. A previous multiomics analysis revealed that decreasing anthocyanin content resulted in the fading of flower color in lilac [8]. Although some candidate genes have been screened, and potential regulatory patterns have been proposed for anthocyanin biosynthesis in lilac flowers [8], their regulatory mechanisms of flower color fading are not yet known.

Anthocyanins, which are derived from the flavonoid pathway, are instrumental in plant ecophysiology processes, such as pollination and coping with various environmental stressors [9, 10]. They also provide key ornamental value by generating the leaf and flower color of such plants as *Padus virginiana* [11], *Paeonia suffruticosa* [12], and *Centaurea cyanus* [13]. The anthocyanin biosynthetic pathway involves multiple genes, some of which have been identified, including the chalcone synthase (*CHS*) gene [14], the flavanone 3-hydroxylase (*F3H*) gene [15], and the UDP-glucose: flavonoid 3-*O*-glucosyltransferase (*UFGT*) gene [16]. Notably, UFGT catalyzes the glycosylation reaction of unstable anthocyanins by forming glucosidic bonds with one or more glucose or rhamnose molecules, thus producing stable anthocyanins [17–20]. Accordingly, being considered a crucial gene in anthocyanin formation, the involvement of *UFGT* has been identified in many plants. For instance, *UFGT* regulates flavonoid metabolism in *Freesia hybrida* [21] and participates in anthocyanin accumulation in *Hibiscus sabdariffa* [22] as well as *Loropetalum chinense* var. *rubrum* [23]. In lilac, although some anthocyanin biosynthesis genes have been identified, including the chalcone isomerase (*SoCHI*) gene and the cinnamic acid 4-hydroxylase (*SoC4H*) gene [24, 25], the relevance of *UFGT* and its role remain unclear, so this urgently needs to be investigated.

**Figure 1:**
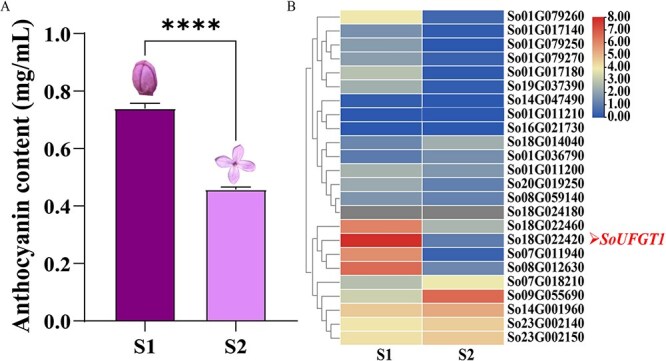
Anthocyanin content and DEGs heatmap at different flower developmental stages in lilac petals. A Anthocyanin content. ^****^P ≤ 0.0001. B The DEGs heatmap. The change in the bar values from small to large indicates expression levels from low to high. S1: bud stage; S2: full-opening stage.

Anthocyanin biosynthesis is regulated by transcription factors (TFs), which bind special *cis*-acting elements in target gene promoters [26]. Several TFs acting on the *UFGT* promoter have been reported. For example, EpMYB1 activates the *Ep3GT* promoter to increase anthocyanin accumulation in *Echinacea purpurea* [27], while CmMYB012 inhibits anthocyanin biosynthesis by suppressing *CmUFGT* expression in *Chrysanthemum morifolium* [28]. Thus, transcriptional regulation of *UFGT* may involve multiple TFs that synergistically contribute to anthocyanin biosynthesis. In red-fleshed apples, not only MdNAC77L markedly enhances *MdUFGT* expression to promote anthocyanin accumulation [29], but also MdNAC1 combines with MdbZIP23 to activate *MdUFGT* [30]. The RcMYB1–RcBHLH42–RcTTG1 complex markedly enhances *RcUFGT* expression in *Rosa hybrida*, which stimulates anthocyanin accumulation [31]. Yet the pattern of *UFGT* regulated by TFs is still unknown in lilac.

In this study, based on the genome and flavonoid metabolic network of lilac [8] during flower color fading, *SoUFGT1* was screened and cloned, and its function was also validated in anthocyanin biosynthesis. Next, SoMYB44 was identified, and its function and regulatory effects on *SoUFGT1* were also determined. Finally, SobHLH130 and SoNAC72 were identified, whose interaction with SoMYB44 was examined to elucidate the regulation mechanism. Accordingly, new insight is gained into the molecular mechanisms responsible for lilac flowers' color fading, which also provides a theoretical basis for improving the molecular breeding of flower color.

## Results

### *SoUFGT1* expression positively correlates with anthocyanin content

To investigate why lilac petal color fades, the anthocyanin contents of petals at different floral development stages were measured. The anthocyanin content decreased as flowers developed, being 60% less at the full-opening stage (S2) than at the bud stage (S1), which led to the fading of purple petals (Fig. 1A). To learn more about the underlying molecular mechanism, the differentially expressed genes (DEGs) in petals were screened at two developmental stages based on transcriptome sequencing (Fig. 1B). Among the DEGs found, the expression pattern of *SoUFGT1* matched the changed anthocyanin content. Thus, *SoUFGT1* would be a pivotal component of anthocyanin biosynthesis in lilac.

### *SoUFGT1* mediates anthocyanin biosynthesis

**Figure 2:**
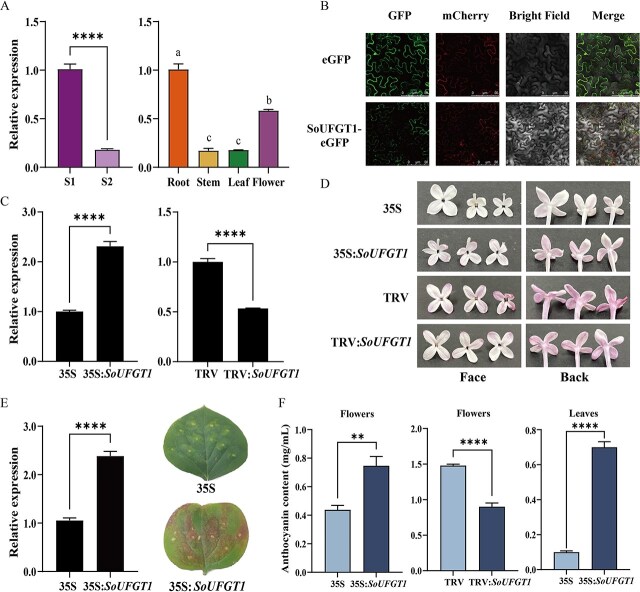
*SoUFGT1* expression pattern, subcellular localization, and functional analysis. A The expression pattern of *SoUFGT1* in spatial and temporal. S1: bud stage; S2: full-opening stage. ****P ≤ 0.0001, different small letters indicated significant difference among different tissues (*P* < 0.05). B Subcellular localization of SoUFGT1 protein. GFP: image taken in the green fluorescence channel; mCherry: image taken in the red fluorescence channel; Bright Field: images taken in bright light; Merge: overlay plots. eGFP: leaves injected with PRI101-eGFP empty vector, as a negative control. SoUFGT1-eGFP: leaves injected with 35S:*SoUFGT1* construct. C Relative expression levels of *SoUFGT1* in S2 stage petals. D Petals phenotype after transient overexpression and silencing of *SoUFGT1*. The left panels represent the face of lilac petals, and the right represent the back. Flowers with light purple or pink color were selected in the transient overexpression experiment; flowers with darker purple color were used in the VIGS experiment. E *SoUFGT1* was overexpressed in leaves. F The anthocyanin content in S2 stage petals and leaves after transient transformation experiments. 35S: PRI101-eGFP empty vector transgenic plants; 35S:*SoUFGT1*: *SoUFGT1* overexpressing plants. TRV: pTRV empty vector; TRV:*SoUFGT1*: *SoUFGT1* silencing plants. ^**^P ≤ 0.01, ^****^P ≤ 0.0001.

The coding sequences (CDSs) of *SoUFGT1* (1146 bp) were cloned from lilac (Fig. S1A). Bioinformatics analysis revealed that SoUFGT1 was a stable hydrophilic protein (Table S2), with high homology to sequences of other plant species, such as *Olea europaea* (Fig. S1B, C). The *SoUFGT1* expression profile was determined during the floral development stages and in different tissues of lilac by real-time quantitative polymerase chain reaction (qRT-PCR), and the results were consistent with the transcriptome sequencing (Fig. 2A). *SoUFGT1* was highly expressed in roots, followed by flowers, at the S2 stage (Fig. 2A). Furthermore, SoUFGT1 was observed to localize on the cytoplasm (Fig. 2B).

To understand the contribution of *SoUFGT1* to lilac anthocyanin biosynthesis, transient overexpression and virus-induced gene silencing (VIGS) experiments in flowers were first carried out by transient agroinfiltration. The *SoUFGT1* expression levels were determined via qRT-PCR (Fig. 2C)*.* Compared with 35S, the expression of *SoUFGT1* in 35S:*SoUFGT1* petals evidently increased, and the petal color got significantly darker (Fig. 2C, D)*.* In stark contrast, after *SoUFGT1* was silenced (TRV:*SoUFGT1*), the petal color instead became lighter (Fig. 2C, D). Similarly, the leaves transiently overexpressing *SoUFGT1* turned from green to red compared to the 35S control (Fig. 2E). As expected, the accumulation of anthocyanins significantly increased in petals and leaves after overexpressing *SoUFGT1*, but vice versa after silencing *SoUFGT1* (Fig. 2F). Collectively, these results indicated that *SoUFGT1* activation was capable of increasing anthocyanin biosynthesis.

### SoMYB44 directly binds to the *SoUFGT1* promoter

**Figure 3:**
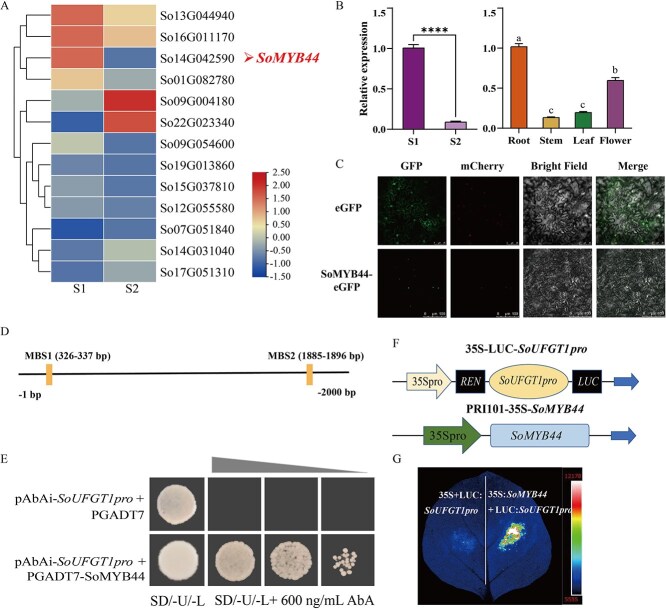
Relationship between SoMYB44 and *SoUFGT1*. A The heatmap of *SoMYB44* expression analysis. The change in the bar value from small to large indicated expression levels from low to high. B Expression pattern analysis of *SoMYB44* in spatial and temporal. S1: bud stage; S2: full-opening stage. ^****^P ≤ 0.0001, different small letters indicated significant difference among different tissues (*P* < 0.05). C Subcellular localization. GFP: images taken in the green fluorescence channel; mCherry: images taken in the red fluorescence channel; Bright Field: images taken in bright light; Merge: overlay plots. eGFP: leaves injected with PRI101-eGFP empty vector, as a negative control. SoMYB44-eGFP: leaves injected with 35S:*SoMYB44* construct. D The MYB binding sites (MBS) of the *SoUFGT1* promoter. E Y1H assay was used to examine SoMYB44 bonds to the promoter of *SoUFGT1*. SD/-U/-L, SD/-Ura/-Leu; AbA^600^ indicated that the concentration of Aureobasidin A was 600 ng/mL. F Construction of double luciferase experimental vector. G Interaction analysis of *SoUFGT1pro* and SoMYB44 by Dual-LUC assay.

Based on time-ordered gene co-expression networks and the transcriptome at the S1 and S2 stage, a MYB TF related to the expression of *SoUFGT1* was selected, and was named here SoMYB44, to explore the regulation mechanism involved (Fig. S2A and Fig. 3A). The expression of *SoMYB44* was notably higher at the S1 than S2 stage, which was consistent with both *SoUFGT1* expression and the anthocyanin content (Fig. 3B). Additionally, higher expression levels occurred in roots and flowers at the S2 stage. (Fig. 3B). The full-length sequence of 1023 bp was then isolated and cloned (Fig. S2B). The phylogenetic analysis showed that SoMYB44 was most closely related to *O. europaea* (Fig. S2C, D) and acted as a TF in the nucleus according to its subcellular localization (Fig. 3C).

To validate the association between SoMYB44 and *SoUFGT1*, the binding sites for the MYB families (MBS) in the *SoUFGT1* promoter region were predicted using the JASPAR website (Fig. 3D). Next, the *SoUFGT1* promoter was cloned and a full-length sequence of 2000 bp obtained. As shown by the yeast one-hybrid (Y1H) results, the strain containing the *SoUFGT1* promoter and AD:SoMYB44 grew normally on SD/−U/−L medium with 600 ng/mL AbA, whereas the *SoUFGT1pro* cotransformed AD did not survive (Fig. 3E). This result preliminarily confirmed that SoMYB44 could bind to the *SoUFGT1* promoter directly. Moreover, to validate this result, the 35S-LUC-*SoUFGT1pro* and RPI101-35S-SoMYB44 vectors were constructed and Dual-LUC assays were then performed *in vivo* (Fig. 3F, G). As displayed in Fig. 3G, the tobacco leaves injected with the 35S-LUC-*SoUFGT1pro* and RPI101-35S*-*SoMYB44 vectors emitted stronger fluorescence than those injected with the empty vector (Fig. 3G). This suggested that SoMYB44 indeed directly bound to the *SoUFGT1* promoter and positively regulated its expression.

### SoMYB44 contributes to anthocyanin accumulation

**Figure 4:**
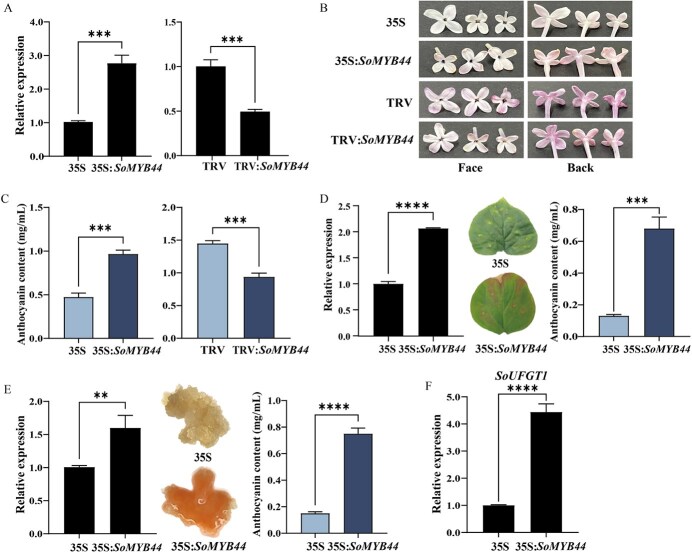
The function analysis of SoMYB44. A Relative expression levels of *SoMYB44* in S2 stage transgenic petals. B Petals phenotype after transient overexpression and silencing of *SoMYB44*. The left panels represent the face of lilac petals, and the right represent the back. Flowers with light purple or pink color were selected in the transient overexpression experiment; flowers with darker purple color were used in the VIGS experiment. C The anthocyanin content in S2 stage petals after transient transformation experiments. D *SoMYB44* was overexpressed in leaves. E *SoMYB44* was overexpressed in calli. F Relative expression of *SoUFGT1* gene after overexpression of *SoMYB44* in S2 stage petals. 35S: PRI101-eGFP empty vector transgenic plants; 35S:*SoMYB44*: *SoMYB44* overexpressing plants. TRV: pTRV empty vector; TRV:*SoMYB44*: *SoMYB44* silencing plants. ^**^P ≤ 0.01, ^***^P ≤ 0.001, ^****^P ≤ 0.0001.

Given its similar expression pattern with *SoUFGT1*, it was plausible that *SoMYB44* also positively regulated anthocyanin biosynthesis. So *SoMYB44* was transiently overexpressed and silenced in lilac flowers to validate its function (Fig. 4A–C). These qRT-PCR results indicated that the expression level of *SoMYB44* in overexpressed petals was about 2.8 times higher than the 35S control, and the silencing efficiency in TRV:*SoMYB44* petals was about 49.3% (Fig. 4A). In terms of phenotype, the petal color of 35S:*SoMYB44* was darker compared to the 35S control, while the petal color of TRV:*SoMYB44* was lighter than the TRV petals (Fig. 4B). By measuring the petals’ anthocyanin content, and the data suggested that 35S:*SoMYB44* petals accumulated an average content of 0.965 mg/mL, about double the 35S control, while that of TRV:*SoMYB44* petals was lower than the TRV control (Fig. 4C). In leaves, compared with the control lines, overexpression of *SoMYB44* led to reddening of the petals, with a significant 5-fold increase in the anthocyanin content (Fig. 4D).

Furthermore, the transient transformation system for lilac calli was constructed. When the concentration of kanamycin and cefalexin was 150 and 100 mg/L, respectively, the calli displayed a browning phenomenon (Fig. S3); so these concentrations were used to screen for transgenic calli. The calli transformed with the 35S:*SoMYB44* exhibited noticeable reddening, and the total anthocyanin content was 1.59 folds higher than the 35S control (Fig. 4E), which further demonstrated the improved effect of SoMYB44 upon anthocyanins. Besides, the *SoUFGT1* expression level increased markedly in 35S:*SoMYB44* petals when compared with the 35S control (Fig. 4F), indicating that SoMYB44 bolstered anthocyanin accumulation by triggering *SoUFGT1* expression.

### SoMYB44 interacts with SobHLH130

**Figure 5:**
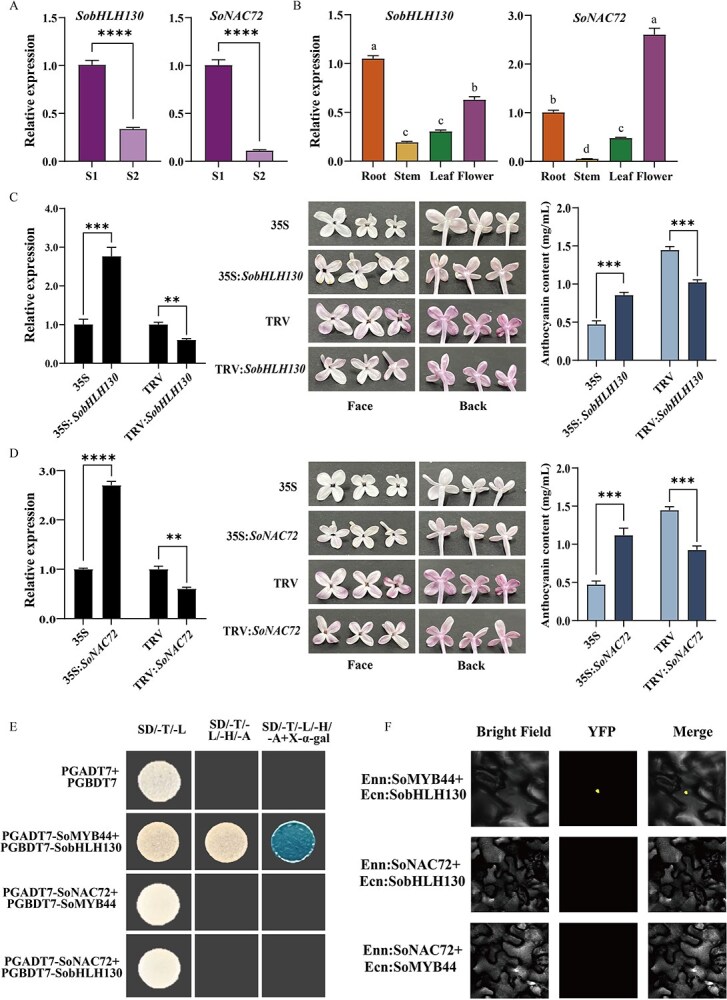
Function of SobHL H130 and SoNAC72, and relationship with SoMYB44. A and B Expression pattern analysis of *SobHLH130* and *SoNAC72* in spatial and temporal. S1: bud stage; S2: full-opening stage. ^****^P ≤ 0.0001, different small letters indicated significant difference among different tissues (*P* < 0.05). C and D The function of *SobHLH130* and *SoNAC72* in lilac petals, respectively. In the S2 stage, the relative expression of genes and anthocyanin content of petals were measured, and the petals' phenotypes were also observed. The left panels represent the face of lilac petals, and the right represent the back. Flowers with light purple or pink color were selected in the transient overexpression experiment; flowers with darker purple color were used in the VIGS experiment. E Interaction analysis among SoMYB44, SobHLH130 and SoNAC72 proteins by Y2H. SD/-L/-T, SD/-Leu/-Trp medium; SD/-L/-T/-H/-A, SD/-Leu/-Trp/-His/-Ade medium; SD/-L/-T/-H/-A + (X-α-gal), SD/-Leu/-Trp/-His/-Ade + X-α-gal medium. F BiFC analysis showing the interaction among SoNAC72, SobHLH130 and SoMYB44 proteins. Bright Field: images taken in bright light; YFP: image taken in the yellow fluorescence channel; Merge: overlay plots. S1: bud stage; S2: full-opening stage. ^**^P ≤ 0.01, ^***^P ≤ 0.001, ^****^P ≤ 0.0001, different small letters indicated significant difference among different floral development stages and tissues (*P* < 0.05).

To better elucidate the mechanism of how SoMYB44 participated in anthocyanin accumulation, its interaction factors were further explored. Based on the obtained transcriptome data from S1 and S2 stages, *SobHLH130* and *SoNAC72*, were selected and likewise validated via a qRT-PCR analysis (Fig. S4A, B and Fig. 5A). Both *SobHLH130* and *SoNAC72* had higher expression in flowers at the S1 stage (Fig. 5A, B); their functions in anthocyanin biosynthesis were examined, using flowers, leaves, and calli (Fig. 5C, D and Fig. S4C, D). After *SobHLH130* and *SoNAC72* were overexpressed, the petal color got darker with the anthocyanin content increasing by about double, but in the silenced petals, the color got lighter with the anthocyanin content decreasing by about 33.33% (Fig. 5C, D). Moreover, *SobHLH130* and *SoNAC72* overexpression caused similar reddening in leaves and calli by augmenting their anthocyanin content (Fig. S4C, D). This set of results confirmed that SobHLH130 and SoNAC72 positively regulated anthocyanin biosynthesis in lilac.

**Figure 6:**
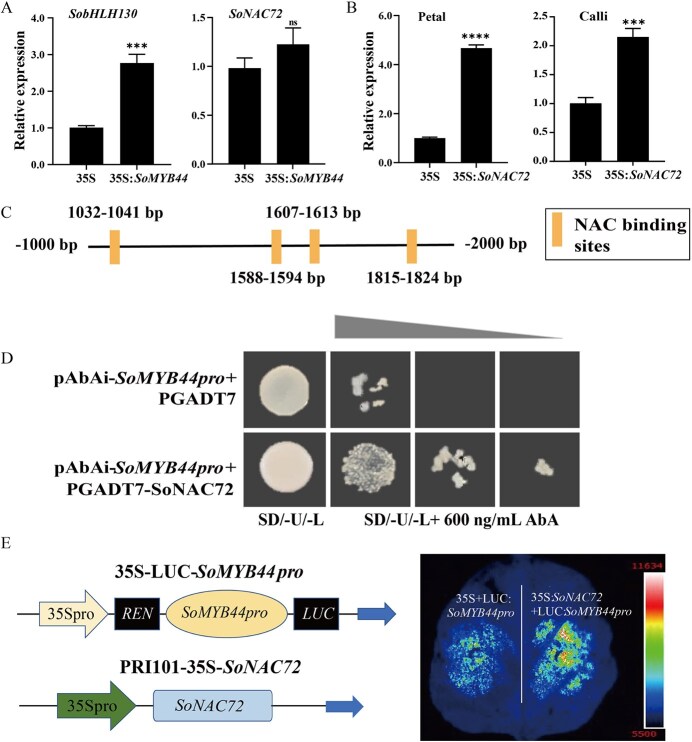
Relationship among SoMYB44 and SoNAC72. A Relative expression of *SobHLH130* and *SoNAC72* after overexpressing *SoMYB44* in S2 stage petals. B Relative expression of *SoMYB44* after overexpressing *SoNAC72* in S2 stage petals and calli, respectively. 35S: PRI101-eGFP empty vector transgenic petals; 35S:*SoMYB44*: *SoMYB44* overexpressing petals, 35S:*SoNAC72*: *SoNAC72* overexpressing petals. ‘ns’ indicates significance at *P* > 0.05; ^***^P ≤ 0.001; ^****^P ≤ 0.0001. C The NAC binding sites of the SoMYB44 promoter (-1000 to -2000 bp). D Y1H assay to examine SoNAC72 combining the promoter of *SoMYB44*. SD/-U/-L, SD/-Ura/-Leu; AbA^600^ indicated that the concentration of Aureobasidin A was 600 ng/mL. E. Interaction analysis of *SoMYB44pro* and SoNAC72 by Dual-LUC *in vivo*.

Next, the interaction of these two TFs and SoMYB44 were examined. Y2H assay indicated that yeast cells transformed with pGADT7:SoMYB44 + pGBDT7:SobHLH130 survived and turned blue in SD/−L/−T/−H/−A media containing X-α-gal (Fig. 5E). Yet the yeast cells cotransformed pGADT7:SoNAC72 with either pGBDT7:SoMYB44 or pGBDT7:SobHLH130 did not grow in SD/−L/−T/−H/−A media (Fig. 5E). These results provided evidence that SoMYB44 and SobHLH130 could interact *in vitro*, while SoNAC72 neither interacted with SoMYB44 nor SobHLH130. The same results were also obtained from bimolecular fluorescence complementation (BiFC) assays done *in vivo* (Fig. 5F). Moreover, SoMYB44 was found to interact with SobHLH130 in the nucleus (Fig. 5F).

### SoNAC72 directly binds to the promoter of *SoMYB44*

In the petals that transiently overexpressed *SoMYB44*, the expression of *SobHLH130* significantly exceeded the 35S control, while *SoNAC72* expression did not confer any significant enhancement (Fig. 6A). Interestingly, the *SoMYB44* expression level increased in both petals and calli after overexpressing *SoNAC72* (Fig. 6B), which suggested that SoMYB44 not only interacted with SobHLH130 but also possibly with SoNAC72. Hence, the promoter elements of *SoMYB44* were analyzed and NAC family binding sites at 1000–2000 bp were detected (Fig. 6C). Furthermore, in Y1H assays, yeast strains containing the *SoMYB44* promoter cotransformed with AD:SoNAC72 grew well on SD/−U/−L media with 600 ng/mL AbA in comparison with those cotransformed with AD (Fig. 6D). In addition, Dual-LUC assays demonstrated that tobacco leaves co-infiltrated with 35S:SoNAC72 and LUC:*SoMYB44pro* exhibited a stronger fluorescence intensity (Fig. 6E), providing further evidence that SoNAC72 was capable of binding directly to the *SoMYB44* promoter to regulate its activation.

## Discussion

The *UFGT* glycosylate hydroxyl groups at C3 and C5 positions increase the stability and diversity of the anthocyanin molecule [32–35], which is essential for the expression of anthocyanins in plants. In *Ficus carica*, when the methylation level of the *FcUFGT3* promoter region is reduced, this enables the high expression of *FcUFGT3* that enhances the production of fig fruit’s purple peel [36]. In *P. suffruticosa*, *PsUF3GT* and *PsUF5GT* have a positive role in anthocyanin biosynthesis of its petals [37]. In this study of lilac, the pattern of *SoUFGT1* expression at two floral development stages was similar to that of anthocyanin content, and overexpression of *SoUFGT1* in green leaves changed their color to red and greatly increased their anthocyanin content; a similar phenomenon was observed in the petals. These results suggest that *SoUFGT1* regulates anthocyanin biosynthesis and accumulation in lilac, which is akin to the role of *UFGT* in *Vitis vinifera* [38], *Prunus mume* [39], *Actinidia chinensis* [40], and *Malus pumila* [41]. The *UFGT* family contains several members, and there is a diverse role in plant growth and development. For example, *UFGT2* contributes to how plants acclimate to abiotic stresses in maize [42], and *ChUFGT* responds to the signal transduction pathway of ABA in *Cerasus humilis* [43]. Moreover, that *SoUFGT1* attained its highest expression level in roots indicated that it could also function in plant growth and development in addition to bolstering anthocyanin biosynthesis.

As a structural gene in the anthocyanin biosynthesis pathway, the expression of *UFGT* is regulated by TFs. As one of the largest TF families in higher plants, MYB is widely reported to regulate the expression of *UFGT* in many species. HaMYB1 directly upregulates the transcriptional levels of *UFGT* to increase the anthocyanin content of *Helianthus annuus* [44]. In *V. vinifera* calli, after overexpressing *VvMYB24*, the calli turned red and the expression level of *VvUFGT* was upregulated [45]. Recently, some novel R2R3-MYB TFs were shown to play a negative regulatory role in anthocyanin biosynthesis. NtMYB2 mainly suppresses the expression of *UFGT* and reduces anthocyanin biosynthesis in Chinese narcissus [46]. Additionally, MYB44 has been found to contribute to differing physiological and biochemical processes among plants. In cucumber, the stability of MYB44 figures prominently in heat stress-induced chlorophyll degradation [47]. In *Brassica napus*, BnaMYB44 participates in carotenoid and abscisic acid biosynthesis by regulating the transcription of zeaxanthin epoxidase genes [48]. Meanwhile, MYB44 is known to confer resistance against bacterial pathogens and to regulate citral biosynthesis [49–51]. So, in order to further understand the molecular regulatory mechanism of *SoUFGT1* in anthocyanin biosynthesis of lilac, based on trancriptome data, *SoMYB44* was isolated and cloned. Through Y1H and Dual-LUC assays, the results shown that SoMYB44 binds directly to the promoter of *SoUFGT1* to activate its expression. Furthermore, the results of function verification demonstrated that SoMYB44 has a positive regulatory effect on anthocyanin biosynthesis and could promote the transcription of *SoUFGT1*. Interestingly, tree peony PsMYB44 is found to regulate anthocyanin biosynthesis, but it is clustered in the MYB44-like transcriptional repressor branch and exerts a negative effect by inhibiting dihydroflavonol-4-reductase (*DFR*) gene expression [52]. Taken together, these findings demonstrate that in different plants, MYB44 performs various functions by targeting different genes, showing its critical role in plant growth and development.

As mentioned earlier, the MBW complex is the most common and important mode of regulating the anthocyanin biosynthetic pathway [53]. In rice, it activates almost all structural genes (*CHI*, *F3H*, and *UFGT*, among others), contributing to anthocyanin biosynthesis, and enhances anthocyanin accumulation in leaves [54]. In this complex, MYB is the core regulatory factor, which is responsible for determining the activation and inhibition characteristics of the structural genes, and it interacts with bHLH to regulate anthocyanin production [55]. ZeMYB9 interacts with a regulator of the IIIf subgroup bHLH TFs, ZeGL3, to promote the biosynthesis of anthocyanins in *Zinnia elegans* [56]. In tomato, SiMYB44 may interact with SiMYC2 to regulate anthocyanin biosynthesis and resistance in response to the JA (jasmonic acid) signaling pathway [57]. Moreover, bHLH may also play a dominant role in MBW complex. In *Lycium ruthenicum*, mutation of a bHLH TF, LrLAN1b, produces white fruit, which is demonstrated to regulate the fruit pigmentation by combining with LrAN2-like, LrJAF13, and LrAN11 [58]. HabHLH1 is positively correlated with anthocyanin content of red sunflower by interacting with HaMYBA [59]. In the present study, *SobHLH130*, having similar expression patterns to *SoMYB44*, was selected to explore its function in anthocyanin biosynthesis and its relationship with SoMYB44. The results showed that SobHLH130 bolstered lilac's anthocyanin biosynthesis and interacted with SoMYB44, both *in vitro* and *in vivo*. The previous researches have found that bHLH130 also mediates anthocyanin biosynthesis in *Rhododendron latoucheae* and improves the drought and heat resistance of *Leucaena leucocephala* [60, 61]. Nonetheless, like MYB44, MdbHLH130 also inhibits anthocyanin biosynthesis in *Malus domestica* by restricting expression of the *CHS* gene under low-nitrogen stress [62]. But, the interaction between bHLH130 and MYB44 or other MYB TFs has been less reported on in plants.

In lilac’s transcriptome, no member of the WD40 family with significant correlation with SoMYB44 got screened, possibly because MYB interacts with bHLH in the MBW complex, but not so with WD40 [63]. Some researches have shown that although WD40 is a member of MBW, it does not regulate structural genes, and it only participates in stabilizing the structure of MBW complex [64, 65]. Interestingly, a member of the NAC family was screened, SoNAC72, whose high correlation with SoMYB44 from the transcriptome suggested its involvement in the biosynthesis and accumulation of anthocyanins in lilac. Therefore, via transient overexpression and silencing experiments, its function was verified. These results suggested that SoNAC72 significantly increased anthocyanin biosynthesis and accumulation in lilac petals, leaves, and calli, which was analogous to the function of PpNAC72 in peach’s (*Prunus persica*) anthocyanin biosynthesis [66]. Besides, NAC72 also plays a positive regulatory role in plants’ tolerance to pathogens, drought, low temperature, and salt stress, as well as in enhancing the sensitivity to ABA [67–69]. In *C. morifolium*, CmNAC25 acts on the promoter of *CmMYB6* to accelerate anthocyanin biosynthesis [70]. In litchi (*Litchi chinensis*), LcNAC002 contributes to anthocyanin accumulation by activating the transcription of *LcMYB1*, an R2R3-MYB TF [71], while LcR1MYB1, as one R1-MYB type factor, is able to interact with LcNAC13 to regulate anthocyanin biosynthesis [72]. Consequently, Y1H and Dual-LUC assays were conducted, whose results suggested that SoNAC72 acted upon the *SoMYB44* promoter and triggered its activity to regulate anthocyanin accumulation; however, no significant interaction was observed between them in the Y2H and BiFC assays. In red-fleshed kiwifruit, AcMADS68 could interact with AcMYB123 and activate the expression of *AcbHLH1* to amplify the regulation effects of the MBW complex in anthocyanin biosynthesis [73]. Correspondingly, in this study, SoNAC72 might enhance the interaction between SoMYB44 and SobHLH130 by activating the expression of *SoMYB44* to promote lilac’s anthocyanin biosynthesis, which would be first to reveal the role of NAC72-MYB44-bHLH130 cascade regulation in anthocyanin biosynthesis of an ornamental plant. Whether SoNAC72 affects the interaction between SoMYB44 and SobHLH130 during the process of anthocyanin biosynthesis and accumulation in lilac requires further validation.

**Figure 7:**
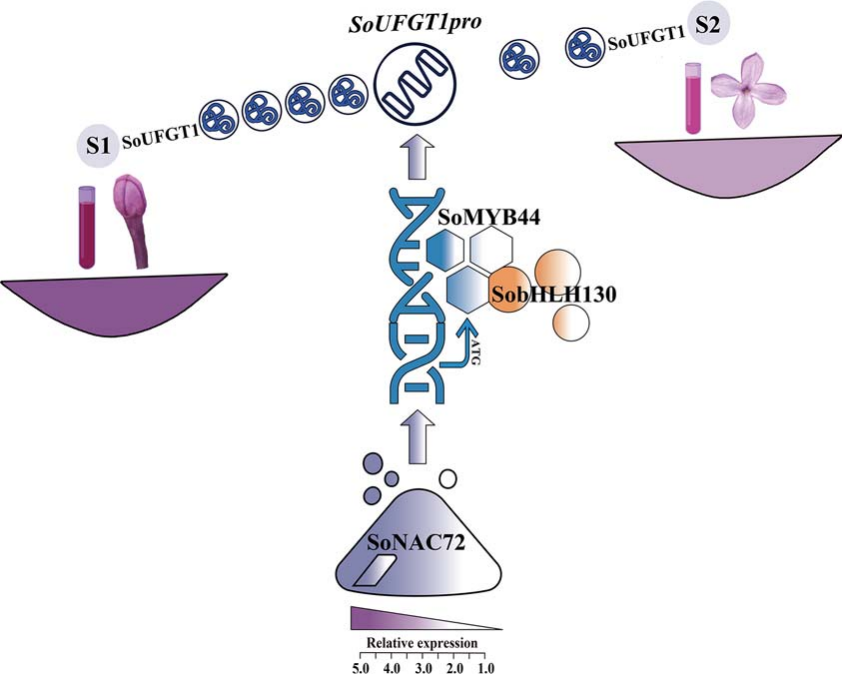
A working model of SoMYB44, SobHLH130, and SoNAC72, regulating *SoUFGT1* to facilitate anthocyanin biosynthesis in *S. oblata*. *SoUFGT1* contributes to lilac anthocyanin biosynthesis; at the same time, *SoUFGT1* was positively regulated by SoMYB44 directly. SoMYB44 not only was regulated by SoNAC72 but also interacts with SobHLH130.

Additionally, the fading of flowers’ color is related to not only the reduction of anthocyanin biosynthesis but also the rate of anthocyanin degradation [74]. Previously, multiomics analysis revealed that decreasing anthocyanin content resulted in the fading of lilac flower color [8]. In this study, it was found that the expression of *SoNAC72*, *SoMYB44*, and *SobHLH130* decreased as the flowers developed, and the regulation of *SoUFGT1* weakened, which reduced the biosynthesis of anthocyanins. As for the degradation of anthocyanins, it might be closely linked to the increases of peroxidase [75] and the changes of pH in vacuoles [76]. Moreover, studies have found that when the level of anthocyanin biosynthesis reduced, the expression level of peroxidase-related genes (such as *PRX*) is elevated [77]. And the pH in vacuoles is also regulated by MBW complex TFs, such as MYB and bHLH [78], but the mechanism of anthocyanin degradation in lilac needed further exploration.

## Conclusion

In summary, SoNAC72-SoMYB44/SobHLH130 module participates in lilac flower color fading. SoMYB44 has a positive role in lilac color formation by ligating to the *SoUFGT1* promoter region to activate latter’s expression, and it was regulated by SoNAC72 and interacted with SobHLH130 in this regulatory relationship (Fig. 7). During the bud stage, TFs had a powerful regulatory effect on *SoUFGT1*, resulting in high anthocyanin content and a dark petal color. As the flowers developed, the expression of TFs decreased, and the regulation of *SoUFGT1* weakened, which reduced the accumulation of anthocyanins (Fig. 7). Altogether, these results partially explain the molecular mechanism of fading petal color and also offer a supply of candidate target genes for molecular breeding of floral color in lilac.

## Materials and methods

### Plant material

*Syringa oblata* cultivated at the nursery of the Beijing University of Agriculture (116°3′14″ E, 40°0′95″ N) was used as the plant material. Petals from the bud stage (S1) and full-opening stage (S2), as defined by Ma *et al.* [8], along with different types of tissue (roots, stems, leaves, and flowers) at the full-opening stage (S2) were collected and promptly frozen at −80°C. The flower branches in the S1 stage were clipped for functional verification testing, and their length was 20 cm. Each sample included three biological replicates.

### Identification and bioinformatics analysis for genes participating in anthocyanin biosynthesis

Overall, 24 *UFGT*, 244 *MYB*, 155 *bHLH*, and 277 *NAC* genes were annotated according to the *S. oblata* transcriptomes, including petals at the two flower development stages (S1 and S2) [8]. Expression heatmaps of those TFs with high expression levels (FPKM [Fragments Per Kilobase of exon model per Million mapped fragments] ≥ 20.0) and significant differences were constructed. After translating the amino acid sequences with DNAMAN software (Lynnon Biosoft, CA, USA), the maximum likelihood method was used to derive trees with 1000 bootstrapped replicates in MEGA 7.0 (MEGA Inc., NJ, USA) to analyze the phylogenetic relationships among the proteins.

### RNA extraction and gene expression analysis

Using the TransZol Up Plus RNA Kit and *TranScript*^®^ One-Step gDNA Removal and cDNA Synthesis SuperMix (TransGen Biotech, Beijing, China), total RNA was extracted from the flowers and then synthesized into cDNA, following the manufacturer’s specifications. The qRT-PCR assay was performed, using *PerfectStart*^®^ Fast Green qPCR SuperMix (TransGen Biotech), to assess the transcript levels of the two flower developmental stages and various tissues at the S2 stage. Relative gene expression levels were based on the expression level of *SoActin* gene, which served as the internal control, obtained by the 2^-ΔΔCT^ calculation. Primers sequences can be found in Supplementary Table S1.

### Transient overexpression in lilac leaves and flowers

The CDSs of *SoUFGT1*/*SoMYB44*/*SobHLH130*/*SoNAC72* were inserted into the PRI101-eGFP vector containing a 35S promoter and respectively renamed 35S:*SoUFGT1*/*SoMYB44*/*SobHLH130*/*SoNAC72*. The construct and PRI101-eGFP (35S, negative control) empty vector were each overexpressed in lilac flowers and leaves by using *Agrobacterium tumefaciens* strain GV3101 (WEIDI, Shanghai, China). For the preparation method of the infection solution, it was referred to Yang *et al.* [79]. The leaves were injected and the lilac flowers were vacuumed for 8–10 min in infection solution. After 24 h of darkness, they were placed in the incubator with 14 h of light and 10 h of darkness, at a temperature of 14°C and 8°C, respectively, which simulated the environmental conditions of lilac flowering to the greatest extent. Three days later, the phenotypes were observed and the anthocyanin contents of their petals at the S2 stage and leaves were gauged.

### Transient silencing in lilac flowers

For the VIGS testing, the *SoUFGT1*/*SoMYB44*/*SobHLH130*/*SoNAC72* fragment (200 bp) was cloned and ligated into the pNC-TRV2 vector (TRV:*SoUFGT1*/*SoMYB44*/*SobHLH130*/*SoNAC72*) then transferred this into GV3101 (WEIDI). Lilac flowers were placed in the mixture of TRV2:*SoUFGT1*/*SoMYB44*/*SobHLH130*/*SoNAC72* and pNC-TRV1 (1:1) vacuumed for 8–10 min. Next, the petals’ phenotypes were observed and their anthocyanin contents measured after flower blooming (S2). The empty pNC-TRV2 and pNC-TRV1 served as the negative control (TRV). Besides, in order to observe the experimental phenotype more clearly, individuals with light purple or pink color were selected in the transient overexpression experiment; while in the VIGS experiment, individuals with darker purple color were used. The corresponding control group was also set up to ensure the accuracy of the experiment.

### Transient overexpression in lilac calli

The seed disinfection followed by the method of *Aesculus turbinata* leaves’ referred to Yamagishi *et al.* [80], but 3% NaClO was used to instead of 10% NaClO and the disinfection time extended to 15 min. Seeds were sown on MS medium [81] containing 3 mg/L 6-BA and 0.1 mg/L IAA. After 1 month, the Top 2 cotyledons of the plant were cut into 1 cm^3^ and laid flat on an induction heating medium (MS plus 7 mg/L 6-BA and 0.05 mg/L IAA).

The 2-week-old calli were infected with *Agrobacterium* containing the 35S:*SoMYB44*/*SobHLH130*/*SoNAC72* plasmid. This infection method was similar to that used for Ponkan mandarin [82]. The ensuing calli were moved to medium containing kanamycin and cephalexin for 20 days of darkness at 24°C. Afterward, they were moved to a 16°C incubator with full light for 10 days to observe phenotypic changes.

### Measurement of anthocyanin content

The total anthocyanin contents of lilac petals, of leaves, and of calli were each determined utilizing the Micro Plant Anthocyanin Assay Kit (Solarbio, Beijing, China). Extraction and measurement of each sample was done three times, independently.

### Subcellular localization

The bacterial solution that respectively contained the 35S:*SoUFGT1*/*SoMYB44*/*SobHLH130*/*SoNAC72* construct, or the PRI101-eGFP empty vector, was injected into *Nicotiana benthamiana* leaves. These treated samples were then observed under a confocal laser scanning microscope (Leica TCS SP5-II, Wetzlar, Germany) after 24 h of 25°C incubation in the dark followed by transfer into light for 2 days.

### Yeast one-hybrid (Y1H) assay

The promoter of *SoUFGT1* (2000 bp) and that of *SoMYB44* (1000 bp) were each cloned (their primers are in Table S1) into the pNC-AbAi plasmid, respectively. After linearizing the construct, it was transformed into Y1H yeast strain (WEIDI) and selected on SD/−Ura medium. Next, *SoMYB44*/*SoNAC72* was ligated into the pGADT7 (AD) vector; this was transformed into Y1H having the pAbAi-bait and selected for positive colonies on SD/−Ura/−Leu + 600 ng/mL AbA (Aureobasidin A) medium.

### Dual luciferase reporter (Dual-LUC) assay

The *SoUFGT1* or *SoMYB44* promoter was attached to the pNC-GreenII 0800-Luc vector. Then, pNC-GreenII 0800-Luc-*SoUFGT1* got cotransformed with 35S:SoMYB44 and pNC-GreenII 0800-Luc-*SoMYB44* got cotransformed with 35S:SoNAC72 (negative control: PRI101-eGFP) into *N. benthamiana* leaves by employing GV3101 (pSoup-p19). After 48 h elapsed, luciferase fluorescence intensity was observed under a CCD camera (Tanon 700, Shanghai, China).

### Yeast two-hybrid (Y2H) assay

The pGBDT7-SoMYB44 and pGBDT7-SobHLH130 recombinant plasmids were each constructed to explore the relationships among SoMYB44, SobHLH130, and SoNAC72. The Y2H tests were completed according to the instruction manuals (WEIDI). Next, the yeast strains carrying pGADT7-SoMYB44 and pGBDT7-SobHLH130, pGADT7-SoNAC72 and pGBDT7-SoMYB44, pGADT7-SoNAC72 and pGBDT7-SobHLH130 were grown on SD/−Leu/−Trp medium, respectively, and then screened the interacting proteins on SD/−Leu/Trp/−His/−Ade medium at 28°C for 3 days. Finally, 4 mg/mL X-α-Gal (Coolaber, Beijing, China) was added to the growing colony to avoid false-positive results.

### Bimolecular fluorescence complementation (BiFC) assay

These pairs of recombinant plasmids—Ecn-SoMYB44 and Enn-SobHLH130; Enn-SoMYB44 and Ecn-SoNAC72; Ecn-SobHLH130 and Enn-SoNAC72—were cotransformed into *N. benthamiana* leaves by employing GV3101 (pSoup-p19). After 24 h of 25°C incubation in the dark followed by transfer into light for 2 days, confocal laser microscopy (Leica TCS SP5-II, Wetzlar, Germany) was used to visualize the YFP signals.

### Statistical analysis

A total of three experiments were carried out in triplicate. Their data were analyzed and visualized in GraphPad Prism v8.4.2 software (La Jolla, CA, USA). The *P* values were determined for Student’s *t* test, where “ns” indicates significance at *P* > 0.05, and ^**^P < 0.01, ^***^P < 0.001, ^****^P ≤ 0.0001. One-way analysis of variance was used, followed by Duncan’s test, with different letters representing significant differences between mean values (*P* < 0.05).

## Supplementary Material

Web_Material_uhae326

## Data Availability

Genes sequences and RNA-Seq data are available from the Short Read Archive under NCBI BioProject ID PRJNA766301. And other relevant data can be found in this manuscript or the supplementary materials.
