# Supplementary material for: SoNAC72-SoMYB44/SobHLH130 module contributes to flower color fading via regulating anthocyanin biosynthesis by directly binding to the SoUFGT1 promoter in lilac (Syringa oblata)
Source: Hortic Res. 2024 Nov 21;12(3):uhae326. doi: 10.1093/hr/uhae326 (PMC11879506; doi:10.1093/hr/uhae326)
Supplement: Web_Material_uhae326 [file web_material_uhae326.docx]

| **Primer** | **Primer sequence** | **Purpose** |
| --- | --- | --- |
| *SoUFGT1* | F: 5'- ATGTCCCTAAAATCCAATTCGC-3' | Gene cloning  Reference primers |
|  | R:5'- TCACTCGTCATGAATGACATTATCAAT-3' |  |
| *SoNAC72* | F: 5'- ATGGGTATTCAAGAAATGGATCCT-3' |  |
|  | R:5'- TCACAGCCTAGACCCCATATTAG-3' |  |
| *SoMYB44* | F:5'- ATGGCGTCTCGTGACG-3' |  |
|  | R:5'- TTAATCGTTGAGCTTACTAATGCCAAT-3' |  |
| *SobHLH130* | F:5'- ATGGATTCCGAAAGAAACACAAAT-3' |  |
|  | R:5'- CTAACAACGTGTTTGATTTGGACC-3' |  |
| *SoACTIN* | F:5'- TGGAATGTGCTGAGAGATGC-3' |  |
|  | R:5'- TGCTGACCGTATGAGCAAAG-3' |  |
| *YGSoUFGT1* | F:5'-TACCACCAGAATAGAGT'-3' | RT-qPCR |
|  | R:5'-AGAAGTATTGCCAGAAG-3' |  |
| *YGSoNAC72* | F:5'-AATCAAGCACAGCCGAGTCA-3' |  |
|  | R:5'-GGCATAGCAGGGGCATACAT-3' |  |
| *YGSoMYB44* | F:5'-AACAAATCGCACCTCAA-3'  R:5'-CCGTCCGACAATAAGA-3' |  |
| *YGSobHLH130* | F:5'-CGGTGTCGCTCTTCAT-3'  R:5'-TAGAGTTGCGGGTTCA-3' |  |
| *Sub-SoUFGT1* | F:5'-AGTGGTCTCTGTCCAGTCCTATGTCCCTAAAATCCAATTCGC-3'  R:5'-GGTCTCAGCAGACCACAAGTCTCGTCATGAATGACATTATCAAT-3 | Subcellular localization |
| *Sub-SoNAC72* | F:5'-AGTGGTCTCTGTCCAGTCCTATGGGTATTCAAGAAATGGATCCT-3'  R:5'-GGTCTCAGCAGACCACAAGTCAGCCTAGACCCCATATTAG-3' |  |
| *Sub-SoMYB44* | F:5'-AGTGGTCTCTGTCCAGTCCTATGGCGTCTCGTGACG-3'  R:5'-GGTCTCAGCAGACCACAAGTATCGTTGAGCTTACTAATGCCAAT-3 |  |
| *Sub-SobHLH130* | F:5'-AGTGGTCTCTGTCCAGTCCTATGGATTCCGAAAGAAACACAAAT-3'  R:5'-GGTCTCAGCAGACCACAAGTACAACGTGTTTGATTTGGACC-3 |  |
| *pRISoUFGT1* | F: 5'- CACGGGGGACTCTAGAATGTCCCTAAAATCCAATTCGCG-3'  R:5'-GGATCCGGTACCCCCGGGCTCGTCATGAATGACATTATCAATGAAACTTC-3 | vector construction |
| *pRISoNAC72* | F:5'-ATGCCCGTCGACCCCGGGATGGGTATTCAAGAAATGGATCCTCTTTCAC-3'  R:5'- TGCTCACCATGGATCCCAGCCTAGACCCCATATTAGTATTACCC-3' |  |
| *pRISoMYB44* | F:5'- ATGCCCGTCGACCCCGGGATGGCGTCTCGTGACGG-3'  R:5'- TGCTCACCATGGATCCATCGTTGAGCTTACTAATGCCAATGC-3' |  |
| *pRISobHLH130* | F:5'-ATGCCCGTCGACCCCGGGATGGATTCCGAAAGAAACACAAATTATAGCC-3'  R:5'- TGCTCACCATGGATCCACAACGTGTTTGATTTGGACCG-3' |  |
| *SoUFGT1pro* | F: 5'- GCGACAGCCAGGTTTTTC-3'  R:5'- CGTTGGCAACTTCATGATGATG-3' | Promoter cloning |
| *SoMYB44pro* | F: 5'-ATGTTGTTCAAATGTGTTGTTCAT-3'  R:5'-TATTAGTACAAGTAAAAGACCTAACT-3' |  |
| JT-*SoUFGT1pro* | F:5'- AGTGGTCTCTGTCCAGTCCT GCGACAGCCAGGTTTTTC -3'  R:5'- GGTCTCAGCAGACCACAAGTT CGTTGGCAACTTCATGATGATG -3' | Construction of recombinant vector |
| JT-*SoMYB44pro* | F:5'-AGTGGTCTCTGTCCAGTCCTATGTTGTTCAAATGTGTTGTTCAT-3'  R:5'-GGTCTCAGCAGACCACAAGTTATTAGTACAAGTAAAAGACCTAACT-3' |  |

Table S1 Primer sequence

| Protein name | Formula | Molecular  Mass/μ | Total number of atoms | Acid-base amino acid | | Aliphatic index |
| --- | --- | --- | --- | --- | --- | --- |
|  |  |  |  | Asp + Glu | Arg + Lys |  |
| SoUFGT1 | C_1959_H_3024_N_508_O_571_S_21_ | 43501.88 | 6083 | 56 | 43 | 82.31 |
| SoNAC72 | C_1722_H_2634_N_480_O_524_S_12_ | 38829.48 | 5372 | 37 | 39 | 62.67 |
| SoMYB44 | C_1311_H_2122_N_386_O_423_S_13_ | 30476.38 | 4255 | 36 | 36 | 75.16 |
| SobHLH130 | C_1587_H_2488_N_496_O_514_S_17_ | 37285.25 | 5102 | 33 | 40 | 54.56 |

Table S2 Physical and chemical properties

**
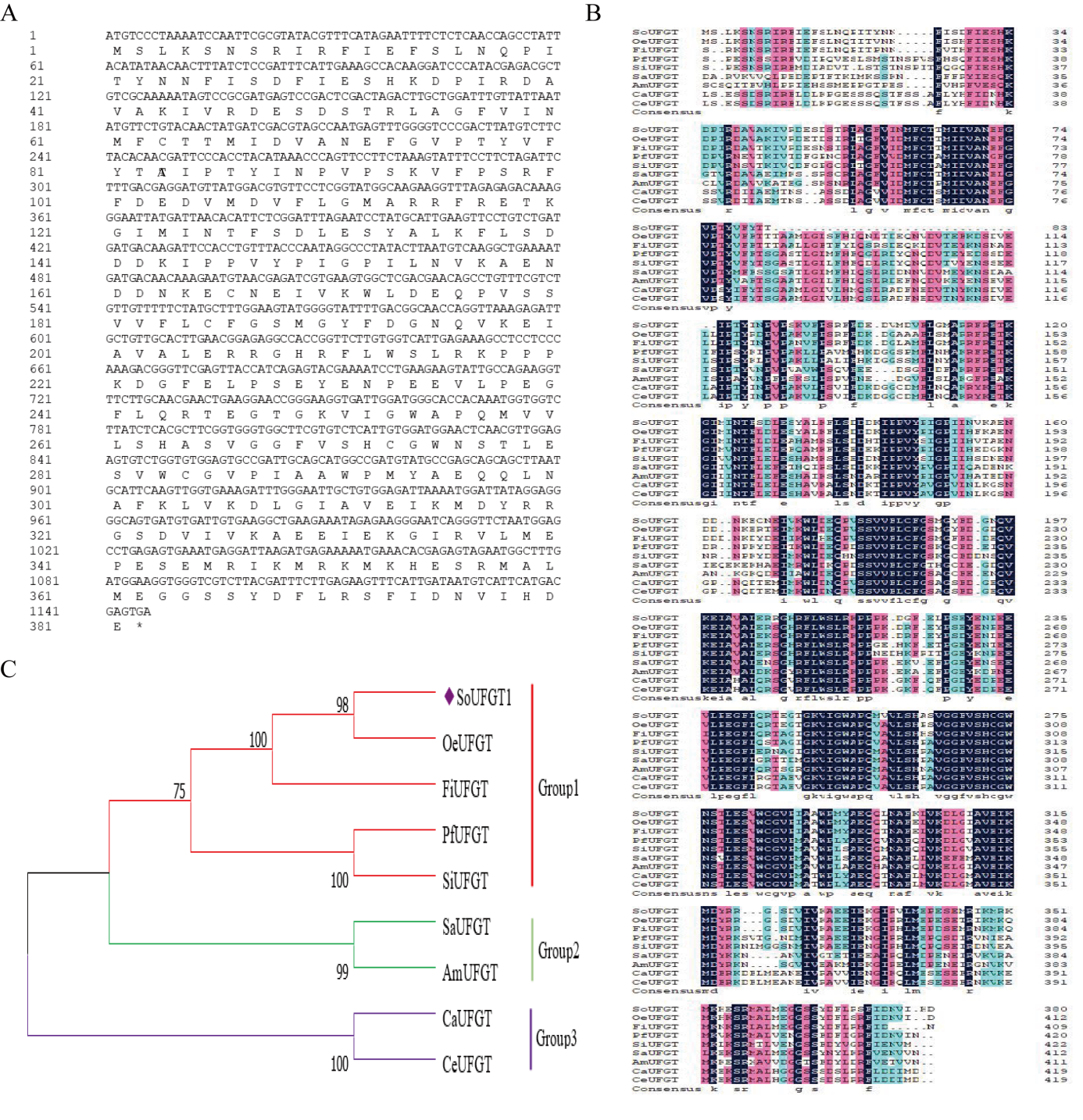
**

**Fig. S1 Bioinformatics analysis of *SoUFGT1*.**

A. Nuclotide acid sequence and amino acid sequence of *SoUFGT1.* B. Multiple sequence alignment of amino acids encoded by *SoUFGT1*. The part of black, red, green shadow respectively represent the homology =100 %, >=75 %, >=33 %. C. Phylogenetic tree of *SoUFGT1* based on amino acid sequences.


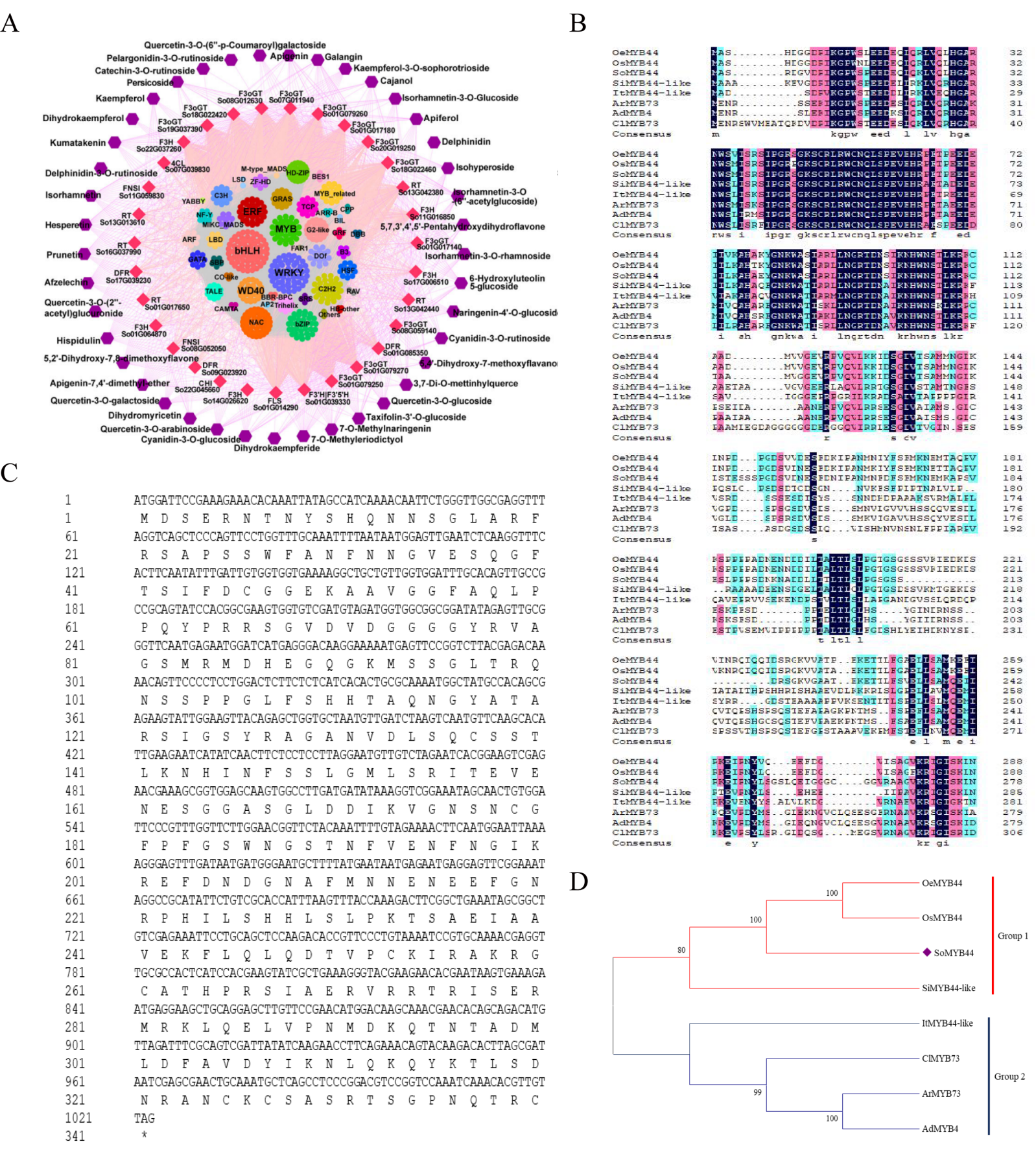


**Fig. S2 Identification and bioinformatics analysis of *SoMYB44*.**

1. Sub-network for flavonoids metabolism by Ma et al. (2022). The network consists of metabolites (purple), structural genes (pink) and TFs from the outside to the inside. B. Nuclotide acid sequence and amino acid sequence of SoMYB44. C. Multiple sequence alignment of amino acids encoded by SoMYB44. The part of black, red, green shadow respectively represent the homology =100 %, >=75 %, >=33 %. D. Phylogenetic tree of SoMYB44 based on amino acid sequences.


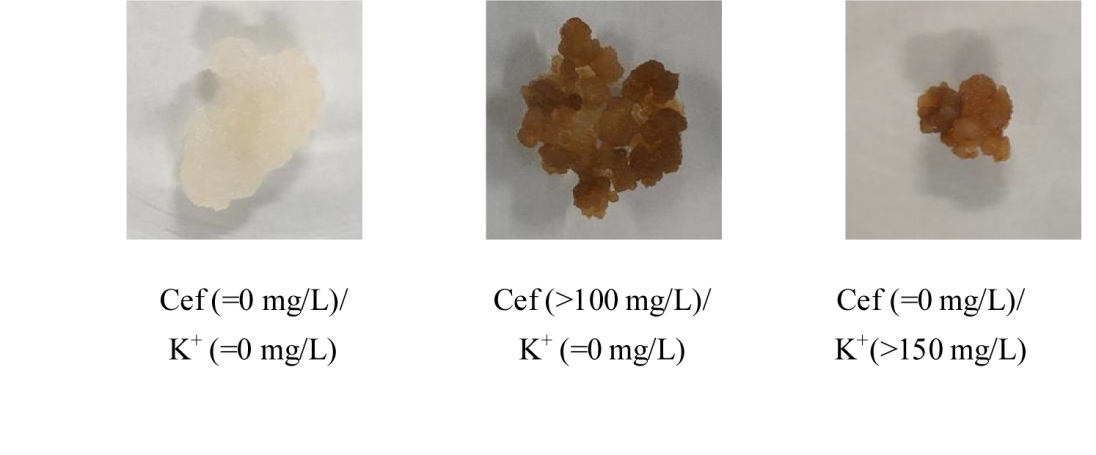


**Fig. S3 Screening of calli growth on different concentrations of antibiotics.**

Cef is short for cephalexin, and K^+^ is kanamycin.


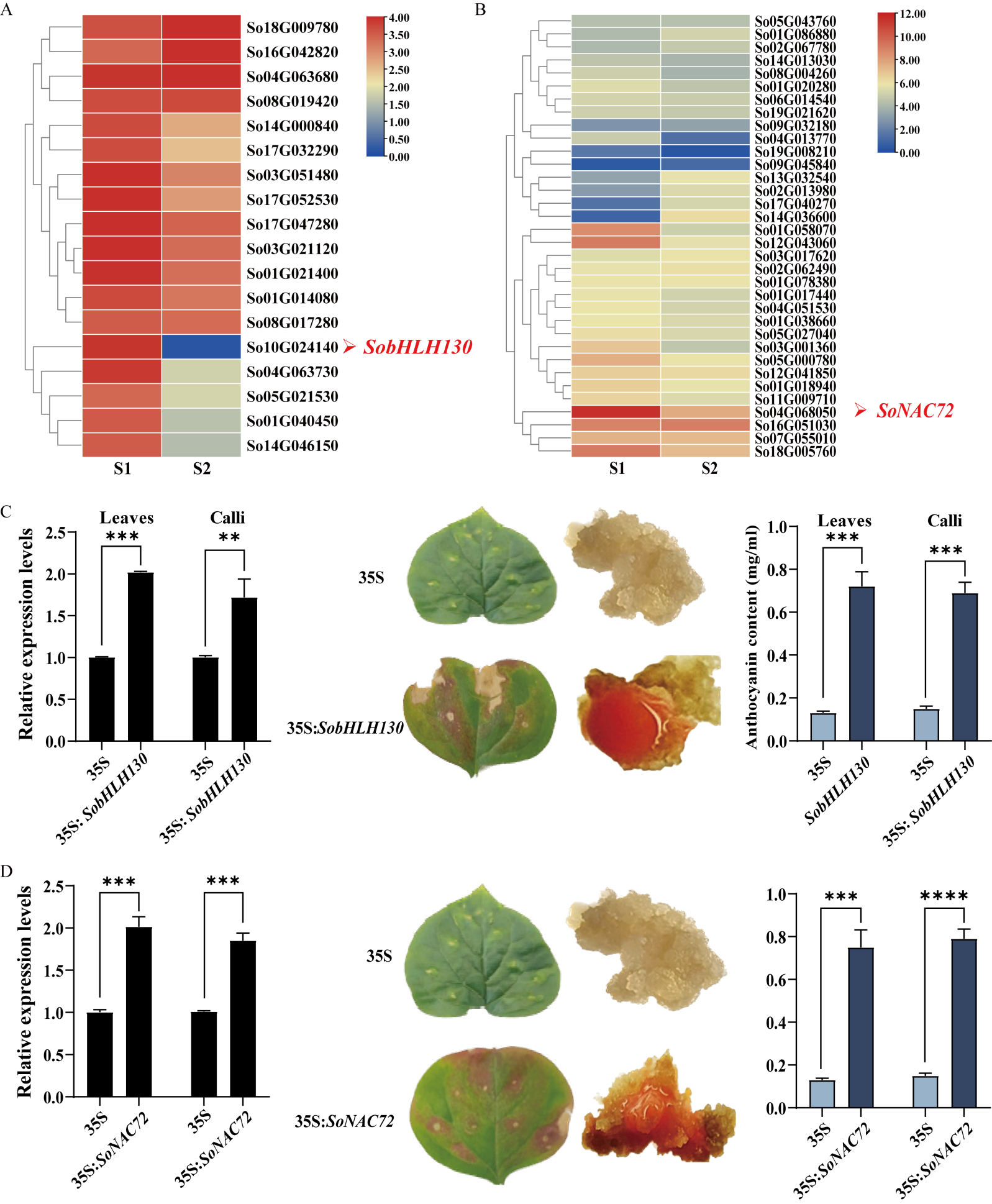


**Fig. S4 Identification and functional verification of SobHLH130 and SoNAC72.** A-B. The heatmap of SobHLH130 and SoNAC72 expression analysis. The change in the bar color from blue to red indicated a change in expression from low to high. C. The function of *SobLHL130 and SoNAC72* in lilac leaves and calli, respectively.
